# RT-qPCR Diagnostics: The “Drosten” SARS-CoV-2 Assay Paradigm

**DOI:** 10.3390/ijms22168702

**Published:** 2021-08-13

**Authors:** Stephen Bustin, Sara Kirvell, Jim F. Huggett, Tania Nolan

**Affiliations:** 1 Medical Technology Research Centre, Faculty of Health, Education, Medicine and Social Care, Anglia Ruskin University Chelmsford, Chelmsford CM1 1SQ, UK; sara.kirvell1@aru.ac.uk (S.K.); tanianolan@btinternet.com (T.N.); 2 National Measurement Laboratory, LGC, Queens Rd, Teddington, London TW11 0LY, UK; Jim.Huggett@lgcgroup.com

**Keywords:** COVID-19, reverse transcription, qPCR, SARS-CoV-2, molecular diagnosis

## Abstract

The reverse transcription quantitative polymerase chain reaction (RT-qPCR) is an established tool for the diagnosis of RNA pathogens. Its potential for automation has caused it to be used as a presence/absence diagnostic tool even when RNA quantification is not required. This technology has been pushed to the forefront of public awareness by the COVID-19 pandemic, as its global application has enabled rapid and analytically sensitive mass testing, with the first assays targeting three viral genes published within days of the publication of the SARS-CoV-2 genomic sequence. One of those, targeting the RNA-dependent RNA polymerase gene, has been heavily criticised for supposed scientific flaws at the molecular and methodological level, and this criticism has been extrapolated to doubts about the validity of RT-qPCR for COVID-19 testing in general. We have analysed this assay in detail, and our findings reveal some limitations but also highlight the robustness of the RT-qPCR methodology for SARS-CoV-2 detection. Nevertheless, whilst our data show that some errors can be tolerated, it is always prudent to confirm that the primer and probe sequences complement their intended target, since, when errors do occur, they may result in a reduction in the analytical sensitivity. However, in this case, it is unlikely that a mismatch will result in poor specificity or a significant number of false-positive SARS-CoV-2 diagnoses, especially as this is routinely checked by diagnostic laboratories as part of their quality assurance.

## 1. Introduction

The reverse transcription quantitative polymerase chain reaction (RT-qPCR) has long-been the research tool of choice for the detection and quantification of a wide variety of RNAs [1]. Nonetheless, the problems associated with reproducibility caused by a range of parameters, including workflow complexity and protocol differences, a diversity of analytical approaches and calibrations, as well as unitage issues, prompted an early recognition of the limitations of the technology as an aide to therapeutic decision-making [2]. Moreover, operator- and reagent-associated variabilities also contribute appreciably to the reliability of RT-qPCR data [3]. There have been numerous publications addressing PCR-related challenges [4,5,6,7,8,9,10,11], culminating in the publication of guidelines to encourage better experimental practices to allow more reliable and unequivocal interpretations of qPCR results [12,13,14]. The uptake of these recommendations has been patchy at best [15,16,17], and until recently, little notice was taken of the significant challenges posed by poor assay designs, a lack of optimisation and validation and variable data interpretation [18]. Even so, one area where RT-qPCR based diagnoses and reproducible quantification guiding therapeutic decisions has been successful is in infectious diseases [19], and RT-qPCR has become the first-line diagnostic test for many different microorganisms [20], including viruses, using a range of chemistries, instruments and protocols [21,22,23,24,25,26].

The emergence of severe acute respiratory syndrome coronavirus 2 (SARS-CoV-2) as the aetiological agent for the coronavirus disease in 2019 (COVID-19) has enhanced the role of RT-qPCR as an essential tool for the early diagnosis of this disease but also highlighted the previously ignored challenges [27]. The initial difficulties have been early and openly acknowledged [28], especially with regards to the divergence of the performances between assays, RT-PCR kits and laboratories [29,30,31] and the emergence of variants refractory to amplification by a commercial assay [32]. However, a substantial body of opinion has emerged, propagated mainly through the press and social media, maligning its use and questioning the validity and utility of RT-qPCR-based testing, in particular. This is, in part, due to criticism of the first such test, which was released with remarkable alacrity only a day or so after the viral genomic sequence was made available and published in a peer-reviewed journal a week later [33]. This test consisted of three assays targeting the genes specifying the RNA-dependent RNA polymerase (RdRp), envelope small membrane protein (E) and nucleoprotein (N). The reverse primer sequence of the RdRp assay contains an incorrect degenerate base S, defined as C or G [34], whereas the SARS-CoV-2 RNA sequence at that position is a T. Whilst the amplification efficiency of the RdRp assay in question can be 100% [35], the quantification cycles (Cq) are higher when compared to a range of other SARS-CoV-2-specific assays [34,35,36,37], affecting the sensitivity, but not the specificity, of the assay [38]. Ironically, the current debate with regards to the interpretation of RT-qPCR test results does not focus on the sensitivity but on the clinical relevance of detecting the virus, often at very low copy numbers. Nonetheless, given the importance of maintaining confidence in the ability of molecular testing to detect SARS-CoV-2 accurately, reliably and sensitively, we dissected the performance of the RdRp assay to determine just how well it performs and to remind the scientific community that mismatches between primers and targets do not necessarily affect the effective performance of an assay.

## 2. Results

### 2.1. One-Step RT-qPCR

**Figure 1:**
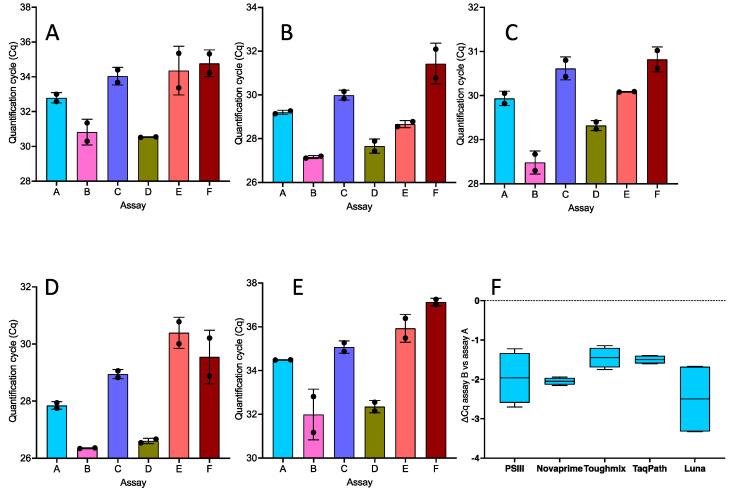
Performance of the six assays listed in Figure 9. Each of the assays was used with different one-step RT-qPCR different master mixes. (**A**) Takara PrimeScript 3. (**B**) GSD Novaprime. (**C**) Quanta 1-step Toughmix. (**D**) ABI TaqPath. (**E**) NEB Luna. Plots are shows as bars with individual Cqs and standard deviations. Each replicate is an independent RT-qPCR reaction. (**F**) Effect of replacing the incorrect original reverse porimer with a correct specific one as shown by the ∆Cq values of assay B compared with assay A for the five master mixes. The box and whiskers plot shows the minimum and maximum ∆Cq values, together with the median (solid line).

The results for the six assays (A–F) amplified with each of the five master mixes are shown in Figure 1A–E. The amplification patterns are similar, with assays B (correct specific R primer) and D (correct specific F and R primers) consistently recording the lowest Cqs. Interestingly, combining the specific F primer ALT Fsp with the mismatched reverse primer resulted in an assay (C) that performed less well than the original assay A (∆Cq = 0.88 (95% CI 0.71–1.05)). Substituting specific primers with wobble primers (assays E and F) also resulted in a poorer performance with all five master mixes. Replacing the original mismatched RdRp SARSr-R primer with the correct, specific primer ALT-Rsp increased the sensitivity of assay B by around four-fold (95% CI 3.05–5.2, with ∆Cqs of −1.96 ± 0.66, −2.05 ± 0.09, −1.45 ± 0.26, −1.5 ± 0.11 and −2.5 ± 0.95 for PrimeScript 3, Novaprime, Toughmix, Taqpath and Luna, respectively (Figure 1F). All Cqs are listed in the Supplementary Data File, Sheet 1.

### 2.2. RT Temperature Effect

**Figure 2:**
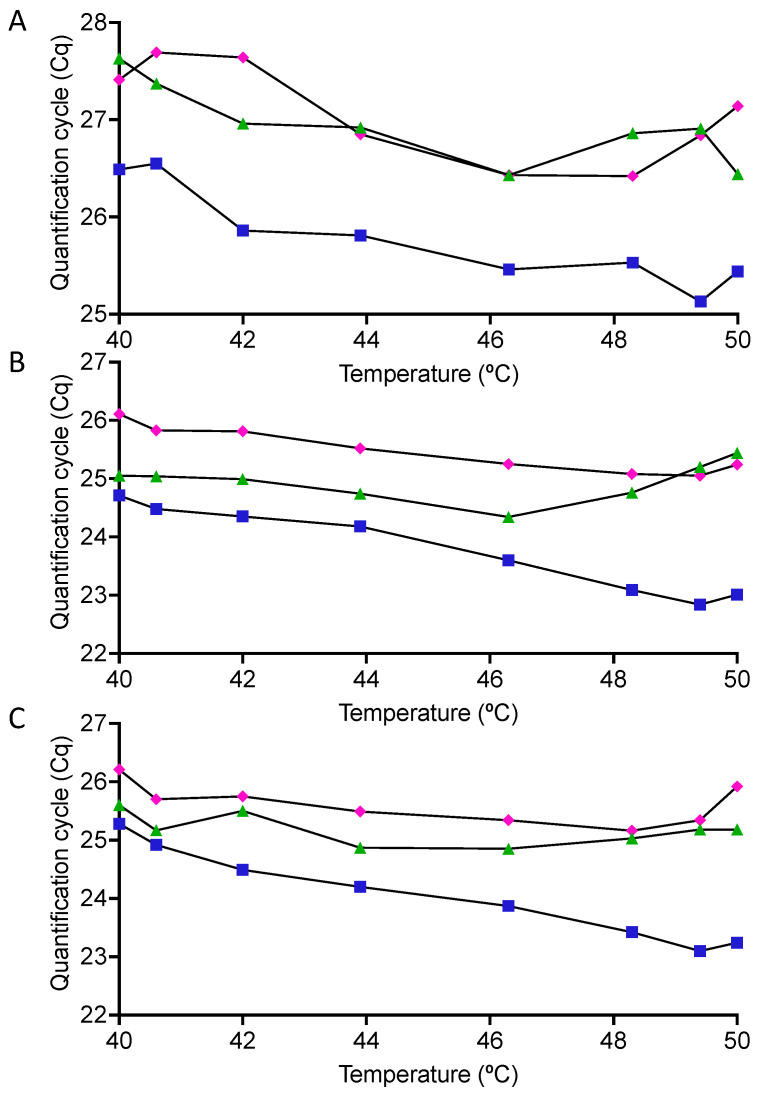
Effect of a reverse transcription temperature gradient on the sensitivity recorded by three assays amplified using different one step RT-qPCR master mixes. (**A**) Assay A with original primers (**B**) Assay B with corrected reverse primer. (**C**) Assay D with specific, correct forward and reverse primers. Quanta Toughmix results are shown with purple diamonds, PCRBio results with green triangles and GSD Novaprime results with blue squares.

The effects of modifying the RT conditions were analysed using three one-step master mixes: all six assays were reverse-transcribed and amplified with GSD Novaprime, whilst PCRBio and Quanta Toughmix were used to assess assays A, B and D. Assay A with the mismatched reverse primer recorded lower Cqs at higher RT temperatures, although the increase in the sensitivity was modest, with the PCRBio assay showing the smallest Cq range at 1.2 and NovaPrime the largest at 1.42 (Figure 2A). Between 42 °C and 50 °C, the Cq range was even smaller; for example, the PCRBio recorded only a Cq difference of 0.5. Assays B and D were affected even less by the RT temperatures, with only the NovaPrime master mix showing a clear effect (Figure 2B,C). Whilst the PCRBio and Quanta Toughmix master mixes recorded similar results, GSD NovaPrime consistently recorded the lowest Cqs. All Cqs, together with those recorded for assays C, E and F amplified by the NovaPrime master mix, are listed in Supplementary Data File, Sheet 2.

### 2.3. Separate RT Primed by Random Primers Followed by qPCR

**Figure 3:**
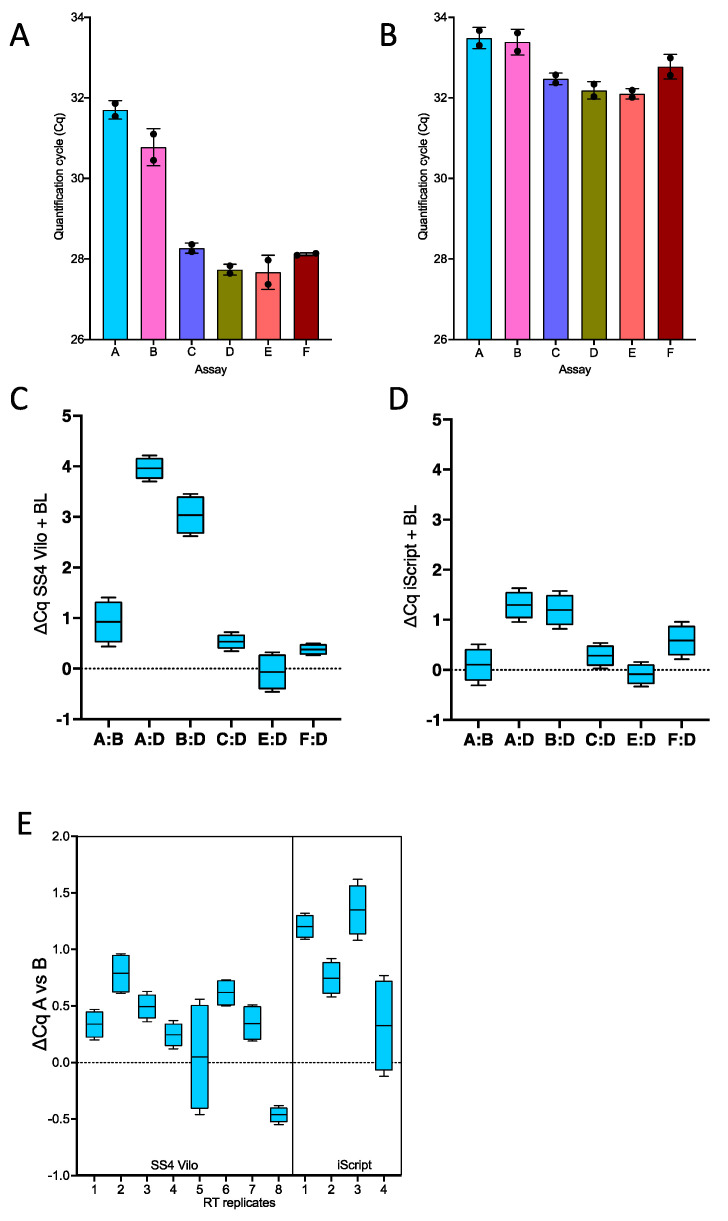
Two step RT-qPCR of the six assays listed in Figure 9 with separate random priming of followed by amplification with SensiFast. (**A**) Scatterplot with bars and SD for Cqs recorded with SS4 RT. (**B**) Scatterplot with bars and SD for Cqs recorded BioRad iScript. (**C**) Plot showing the frequency distribution of the SS4 ∆Cq data, together with the median (solid line). A:B compares the original assay to the one with a correct specific reverse primer. The others compare each of the assays against D, which uses specific forward and reverse primers. (**D**) Plot showing the frequency distribution of the iSCript ∆Cq data, together with the median (solid line) for the same assay combinations. (**E**) Plot showing the frequency distribution of the 2 × 4 replicate SS4 and for replicate iScript ∆Cq data, together with the median (solid line).

The relative contributions of the RT and qPCR steps were further investigated by reverse-transcribed RNA using random primers with Superscript IV Vilo (SS4) or iScript and subjecting the cDNA to qPCR amplification. This resulted in a rather different amplification pattern compared with each other, as well as the one-step methodology for both RTs (Figure 3A,B). First, the Cqs recorded by SS4-transcribed cDNA were consistently lower than those from iScript. Second, assays A and B gave broadly comparable results, with the ∆Cqs similar at 0.93 (95% CI 0.26–1.59) for SS4 (Figure 3C) and 0.1 (95% CI −0.44 to 0.63) for iScript (Figure 3D). Third, assays A and B performed much worse with SS4 than assay D, recording a ∆Cq_A:D_ of 3.96 (95% CI −0.44 to 0.63) and ∆Cq_B:D_ of 3.04 (95% CI 2.42–3.66). In contrast, the ∆Cqs with cDNA transcribed with iScript were 1.30 (95% CI 0.85–1.74) and 1.19 (95% CI 0.69–1.69), respectively. Finally, assays C, D, E and F recorded similar results with both cDNAs, suggesting that the presence of wobble bases in the primers did not affect the PCR reaction.

The near-equivalence in the results obtained for assays A and B was corroborated by repeating the RT reactions for both RTases using a different RNA sample, this time carrying out eight separate RT reactions with SS4 and four with iScript. (Figure 3E). The SS4 results (∆Cq = 0.3 (95% CI 0.16–0.45)) were equivalent to the previous experiment, whereas the iScript results showed a slightly larger ∆Cq (∆Cq = 0.91 (95% CI = 0.65–1.16)), but nevertheless, they were in line with the previous results. All the Cqs are listed in Supplementary Data File, Sheet 3.

### 2.4. qPCR Temperature Effect

**Figure 4:**
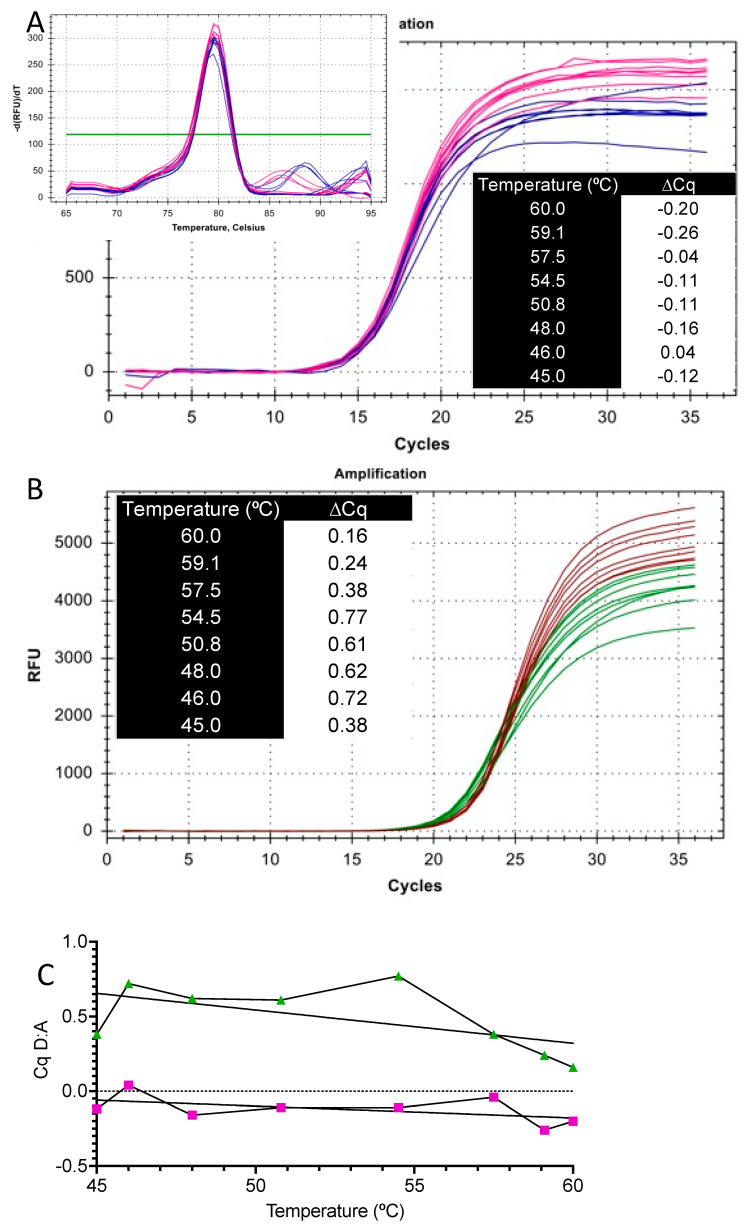
Effect of annealing temperature on RT-qPCR. cDNA synthesised by BioRad iScript was used to amplify assays A and D with Bioline SensiFast master mix. (**A**) SYBR-green reported amplification plots and ∆Cq values for assays A (blue) and D (pink), with the melt curves shown in the insert. (**B**) Amplification plots and ∆Cq values for assays A (green) and D (brown). (**C**) Plot of ∆Cq vs. temperature with trend lines for the SYBR Green (pink) and probe-based (green) assays.

Compared with SS4, the ∆Cqs between assays A and D with the iScript-derived cDNA were unexpectedly small (3.83 ± 0.31 vs. 0.96 ± 0.34). Hence, the qPCR analysis of both assays was repeated with another sample of cDNA synthesised by iScript. The first three cycles were carried out using a 45.0–60.0 °C annealing/polymerisation gradient, followed by a standard amplification at 60 °C, with either SYBR Green (Figure 4A) or RdRp_SARSr-P2 (Figure 4B) as the reporters. In both cases, the Cqs recorded by assay A were comparable to those for assay D (Figure 4C), and, notably, despite the supposedly incompatible melting temperatures of the primers, both assays amplified equally well at all the temperatures, even above 58 °C, with melt curves showing single amplicons (Figure 4A, insert). All the Cqs are listed in Supplementary Data File, Sheet 4.

**Figure 5:**
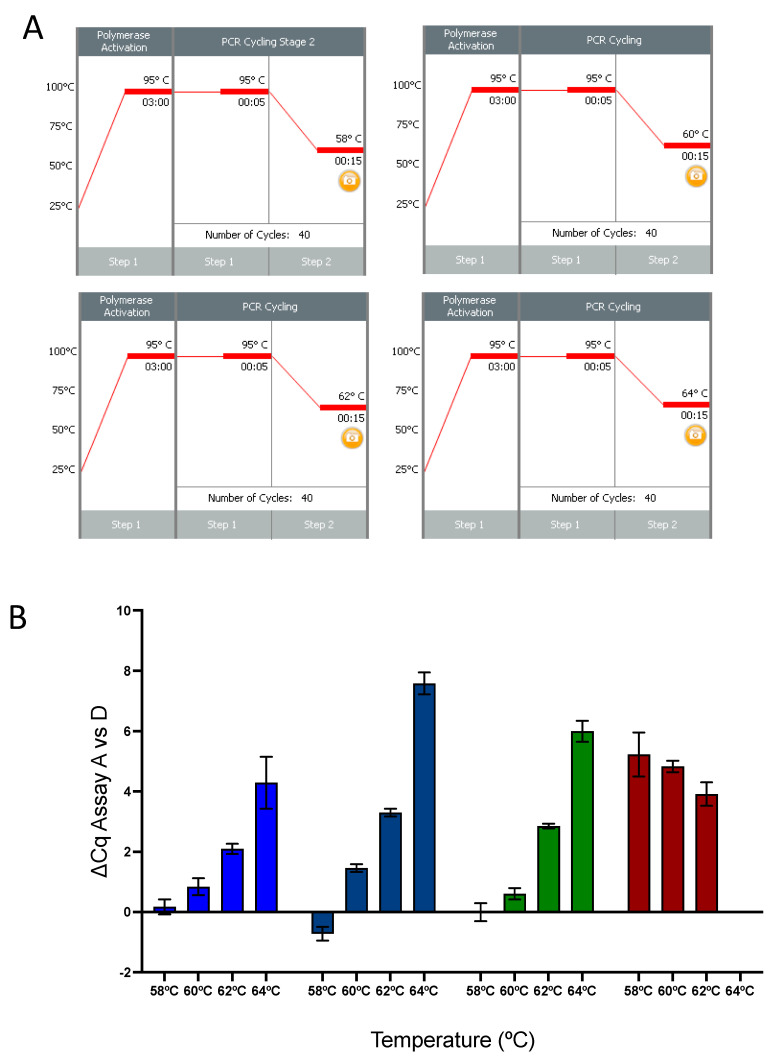
Influence of master mix on qPCR performance of assays A and D with cDNA synthesised by BioRad iScript. (**A**) Protocols of the four annealing/polymerisation conditions. (**B**) Plots of the ∆Cqs between assays A and D, carried out in duplicate at the four temperatures recorded with PrimerDesign (blue), Roche (turquoise), Quanta (green) and ABI (brown) master mixes. The error bars show standard deviations.

The performance of assays A and D at different annealing/polymerisation temperatures (58 °C, 60 °C, 62 °C and 64 °C, Figure 5A) was further investigated using four additional master mixes and a different qPCR instrument. The results for assay A were equivalent to those of assay D at an annealing/polymerisation temperature of 58 °C for three of the master mixes, with the PrimerDesign, KAPA and Quanta master mixes giving similar results, with ∆Cqs_A:D_ of 0.17 ± 0.25, −0.72 ± 0.23 and 0.01 ± 0.30, respectively (Figure 5B). The Thermo Fisher master mix performed significantly worse (∆Cq = 5.23 ± 0.73). Increasing the annealing/polymerisation temperature to 60 °C resulted in a deterioration of the performance of assay A relative to that of assay D, especially for the PrimerDesign master mix, a trend exacerbated by further increases to 62 °C and 64 °C, although assay A was able to amplify its target even at 64 °C. The Thermo Fisher master mix continued to be the worst performer and failed to amplify either assay at 64 °C. All Cq values are listed in Supplementary Data File, Sheet 5.

### 2.5. Comparison of Mismatched and Corrected RdRp Probes

**Figure 6:**
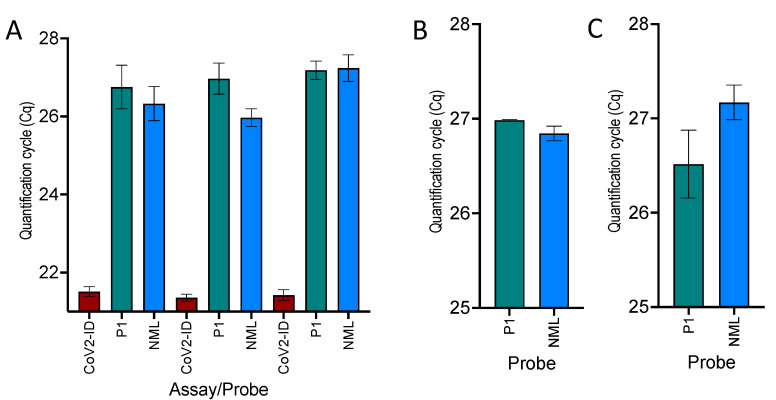
Effect of mismatches between probe and target sequence. Column bar charts are shown plotted as the mean Cq± SD. RdRp_SARSr-P2 results are shown in green, those obtained with NML P1 dg in blue. (**A**) Cqs from three replicate PCRBio 1-step RT-qPCR assays demonstrate that whilst the RdRp assay is less sensitive than CoV2-ID, the mismatched probe records approximately the same Cqs as the non-mismatched one. (**B**) Cqs were recorded using cDNA reverse transcribed by UltraScript and amplified by SensiFast qPCR master mix. (**C**) Cqs were recorded using cDNA reverse transcribed by SS4 and amplified by SensiFast qPCR master mix.

The performance of assay G, which uses the original mismatched RdRP_SARSr-P1 probe, was compared to assay H, which substitutes that probe with the corrected ALT-P1dg sequence. The performance of both was compared to that of a published SARS-CoV-2 assay (CoV2-ID) [39]. Three replicate assays using the PCRBio one-step RT-qPCR master mix recorded equivalent Cqs, with G:H ∆Cqs of 0.42 ± 0.71, 1.01 ± 0.50 and −0.05 ± 0.45, respectively (Figure 6A). As expected, and in line with the previously reported lower sensitivity of this assay, both assays were less sensitive than CoV2-ID, with average ∆Cqs of 5.09 ± 0.64 and 5.54 ± 0.75 for G and H, respectively. Repeating the comparison between assays G and H using two-step RT-qPCR assays with cDNA synthesised by Ultrascript (Figure 6B) or SS4 (Figure 6C) and amplified using Bioline’s SensiFast qPCR master mix confirmed the equivalence of the two probes, indicating that the two mismatches did not affect the ability of the probe to bind to the PCR amplicon. The Cqs are listed in Supplementary Data File, Sheet 6.

### 2.6. Effect of Increased Reverse Primer Concentration

**Figure 7:**
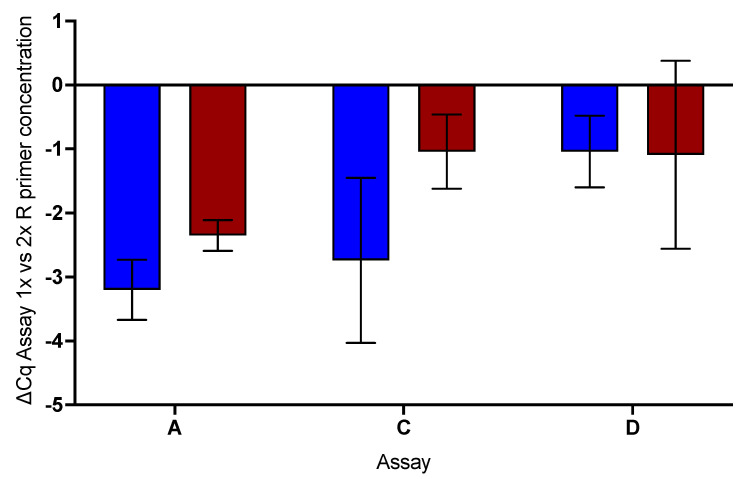
Effect of doubling R primer concentration for RT-qPCR analysis of RNA with assays A, C and D. The lower Cqs recorded by the higher primer concentration are apparent for assays A and C carried out using PrimeScript 3 (blue), compared with assay D, where doubling the concentration of NML R sp made little difference. Assay A was also enhanced with the PCRBio (brown) master mix, whereas assays C and D were not. The error bars show the standard deviations recorded by two independent replicate reactions carried out with each assay.

Finally, the potential for increasing the efficiency of the RT step was investigated by doubling the concentrations of the reverse primers in assays A, C and D and carrying out RT-qPCR assays with the PrimeScript 3 and PCRBio one-step master mixes. This resulted in lower Cqs for assays A and C, especially with the PrimeScript master mix (Figure 7). There was no real effect on the Cqs recorded by assay D run with specific primers. The Cqs are listed in Supplementary Data File, Sheet 7.

## 3. Discussion

This investigation clarifies and restates a number of issues with regards to the Charité RdRp assay:

1. The single base mismatch in the reverse primer reduces the sensitivity of the assay by affecting the RT step.
2. The qPCR step is less affected by the primer mismatch than has been suggested.
3. In one-step RT-qPCR reactions, specific primers perform better than those that incorporate wobble bases.
4. The two mismatches in the RdRp_SARS-P1 probe do not affect the performance of the assay.
5. Although there is a significant difference in the Tm between the forward and reverse primers, our data show that the RdRp assay performs reliably at a broad range of annealing temperatures and well above the calculated Tm for the R primer.

The performance of this assay has been evaluated previously in several publications and shown to result in higher Cqs and a reduced sensitivity when compared with assays targeting other viral genes [34,35,36,37,38]. The actual reduction, however, is not clear. This is because the ∆Cqs vary considerably between reports, and the protocols used are also significantly different (Supplementary Data File, Sheet 8). Critically, there is no agreement on which of the most commonly used assays is the most sensitive one. The overall quality of a molecular diagnostic test is dependent on an optimised, complete workflow starting with sample selection and collection and ending with appropriate data, rather than individual components of the workflow [31]. The performance differences of the various SARS-CoV-2 RT-qPCR kits are due to both the different viral sequences being targeted and, also, the different reagents and master mix formulations, including the kit production quality [30]. Certainly, the conclusion from one of the papers is worth repeating that “thanks to this [the Charité RdRp] assay an important number of COVID-19 diagnoses were made, which contributed to limiting the spread of the outbreak” [38].

The RdRp-SARSr-R mismatch might be expected to affect either the RT or the PCR, depending on whether the cDNA protocol is a one-step RT-qPCR protocol using specific RT primers or a two-step protocol using random priming. In vivo [40] and in vitro [41,42,43] studies established long ago that reverse transcription is a 3′-mismatch-tolerant process, a conclusion supported by more recent analyses [44]. Another study has shown that as many as four internal mismatches have no effect on RT efficiency [45], and mismatched primers are efficiently extended by Avian Myeloblastosis Virus-RT [46], again confirmed by a more recent report [47]. Importantly, the effect of a mismatch varies up to seven-fold depending on the master mix used [47]. Lastly, once a mismatched primer is reverse-transcribed into a cDNA template, both are fully complementary, and no dramatic negative effect would be expected for the subsequent PCR.

The data presented here support these inferences and extend them to demonstrate that the extent of the mismatch-associated effect depends on three factors: (i) the choice of RT and (ii) RT-qPCR amplification strategy, as well as (iii) the selection of RT-qPCR master mix reagents. As demonstrated, the use of one-step protocols results in an average variability of around four-fold across the five master mixes for the mismatch assay compared with the corrected assay. Replacing the wobble bases altogether had no beneficial effect. Combining a specific forward primer with the mismatched reverse primer had a slightly deleterious effect, though the corrected wobble R primer performs the least well. However, there are reagent-dependent differences; for example, assay E performs better than assay A with the GSD Novaprime master mix.

The RT temperature gradient results shed some more light on the effects of the R primer mismatch on the RT step within a one-step RT-qPCR assay. Lower annealing temperatures have little effect on the RT efficiency for all three assays, although, again, there are clear differences between the reagents, with the GSD Novaprime master mix recording lower Cqs and performing better at the higher temperatures. However, clearly, the mismatch is not significantly destabilised at higher RT temperatures, and lowering the RT temperature does not restore an efficient cDNA synthesis from the mismatched primer.

Whereas assay A consistently performed poorly compared to assay B in the one-step RT-qPCR reactions, a two-step approach involving a separate RT step that includes random primers minimised the effect of the mismatch, a result observed for both RTs tested. This was reproducible with the amplification of multiple independent RT replicates. Interestingly, there was an RT-dependent difference in the ∆Cqs between A and D. The reduced sensitivity can be ameliorated by adjusting the experimental conditions, most obviously by increasing the concentration of the mismatched reverse primer. These results differed from those reported elsewhere [37], but whereas these authors did not actually increase the primer concentrations above those originally reported, the concentration of RdRp_SARSr-R in this study were doubled to 1.6 μM.

The cDNA obtained using random primers incorporates any initially mismatched nucleotides, and although the efficiency of the extension depends on how efficiently a primer hybridises onto its complementary target sequence [48] and how effective Taq polymerase binds to both [49], the consequences of the mismatches during the PCR reaction are not straightforward [50]. A series of studies has established that the effects of the mismatches are variable and depend on the sequence context [49,51], the nature of the mismatch [52], the reaction conditions [53,54] and the polymerase [55], as well as the primer length [56]. Single mismatches, especially when located well away from the 3′-end of the primer, generally have a small effect on PCR amplification [57,58,59]. An internal G:T mismatch is the least affected, and lowering the annealing temperature improves the primer extension efficiency of almost all single mismatch types occurring at positions other than the last 3–5 bases from the primer 3′-end [60]. This is, of course, relevant, since not only is the mismatch 15 nucleotides from the 3′-end of the reverse primer, but it results in the most favourable G:T pairing expected to have a minimal effect on the qPCR assay. This deduction is borne out by the comparable performances of assays A and D over a wide temperature range in the qPCR reactions carried out with the first three cycles, involving a 45.0–60.0 °C annealing/polymerisation gradient.

An analysis of the compatibility of the primers and probe suggests that they are not a good match by the accepted design criteria. One unanticipated feature of the two primers is their disparate Tm, with the forward primer predicted to have a significantly higher Tm than the reverse one. Conventionally, qPCR uses equimolar concentrations of two primers with similar Tm. However, this is not necessary for an efficient PCR, as demonstrated by Linear-After-The-Exponential (LATE)-PCR [61], where limiting the concentration of one primer results in a decreased Tm for that primer compared to the one that is in excess [62]. The optimal annealing temperature, which is where most or all of the primers are bound to their target, is likely to be quite different from the Tm, which is defined as the temperature where 50% of the primers are bound to their target. The performance of the assay across a range of annealing conditions above the predicted Tm shows that the choice of master mix is important and that even a widely mismatched primer pair can result in efficient amplification. Interestingly, mismatches in the probe that might be expected to affect its performance by reducing its Tm have little effect, as shown by a comparison of the mismatched original probe with a corrected version. It is expected that sufficient probe binds to the template prior to the primer extension, maintaining adequate exonuclease digestion of the probe.

If use of a mismatched reverse oligonucleotide results in less efficient cDNA synthesis priming, one way of ameliorating this issue could be to increase its concentration, thus improving the cDNA yield and, hence, the sensitivity of the assay. This is indeed possible, although doubling the concentration of the mismatched RdRp_SARSr-R primer improved the performance of assay A and, to a lesser degree, that of assay C without significantly affecting assay D.

Lastly, no discussion of a diagnostic test would be complete without an acknowledgement that, no matter how sensitive, accurate and reliable an RT-qPCR assay might be, appropriate interpretation of the results is an equally important, final component of the informed, clinical decision-making process. Whilst RT-qPCR can provide a measure of the abundance of SARS-CoV-2 RNA above a technical threshold, it remains unclear how that abundance, usually expressed as a Cq value, translates to infectiousness or the need to implement rigorous public health measures. This is exemplified by the wide Cq variability observed after the amplification of the same RNA sample with a commercial primer and probe (PrimerDesigns Coronavirus Genesig assay) and nine different commercial master mixes (Supplementary Figure S1). This is expected, and has been previously discussed, but adds weight to the argument that reporting unqualified Cqs in the context of testing for SARS-CoV-2 is meaningless.

In theory, the use of RT-qPCR testing to determine the SARS-CoV-2 viral load should be useful for the clinical management of individuals and the assessment of their need to self-isolate, as well as the launch of contact tracing. However, although samples with lower Cq values generally have more viral RNA than those with higher Cq values [63], the clinical relevance and precision associated with those differences has not been determined [64], and even the meaning of “high” Cq is undefined. The inconsistencies in using Cq cut-off values were demonstrated by a publication reporting no positive culture growth in samples with Cq > 24 [65]; another reporting a positive culture in samples with Cqs of 34 and a negative culture with Cqs of 22 [66] and yet another report of no positive culture above a Cq of 34 but 12% positive at 33, 50% at 21, 28% at 30, 70% at 29 and 53% at 27 [67]. Crucially, a single raw, not normalised Cq value is not a quantitative result. At the very least, a Cq informed quantity would have to involve normalisation using some marker of the cell mass or the mucosal surface [68] and, crucially, be considered in a clinical context [69]. Sadly, the kind of results where one group reports no correlation between the Cq values and severity of the disease or mortality [70] and another that claims that the Cq value predicted the disease severity and survival [71] is quite the norm. In practice, there are several reasons that Cq values continue to have limited use in clinical settings. First, a positive PCR test alone does not correlate with infectivity, and currently, there is no standard measure of a viral load in clinical samples. Second, numerous variables have a bearing on the viral load, and the patient outcomes are determined by an additional set of variables such as comorbidities and age. Third, the Cq values are relative, as they are affected by sample collection and processing [72], the targets, reagents, assays [73] and instruments [74]. Fourth, there is a statistical uncertainty around any Cq value, which, for RT-qPCR, can exceed 3 Cqs under some circumstances, such as when the template is degraded or present at a low copy number. Fifth, the Cq values and a lower viral load may not be directly proportional, because inhibitory factors within samples may cause a later amplification [75]. Sixth, it has been known for a long time that the testing of the same sample by different laboratories can result in a huge variability in the recorded Cq values. Consequently, it is not feasible to dictate a universal Cq to demarcate positive from negative test results [76], even if the Cq values are translated as units of a pathogen load based on a standard curve. Until suitable, clinically validated standards are available, it is not possible to correlate the data obtained from different testing facilities.

In conclusion, the reverse primer mismatch in the RdRp component assay of the first published SARS-CoV-2 test affects the performance of that assay. In contrast, the mismatched probe has no appreciable effect on the assay sensitivity. Importantly, it is possible to ameliorate the effects of the primer mismatch through a combination of optimal RT, reagents and protocols. Whilst we would continue to stress that it is important to design assays carefully from the start, our findings hold an important lesson for RT-qPCR assays in general, as they highlight the flexibility and robustness of this methodology, where even a suboptimal design can be rescued by intelligent optimisation.

## 4. Methods and Materials

Details of manufacturers and suppliers are listed in the Supplementary Data File, Sheet 9.

### 4.1. Primers and Probes

**Figure 8:**
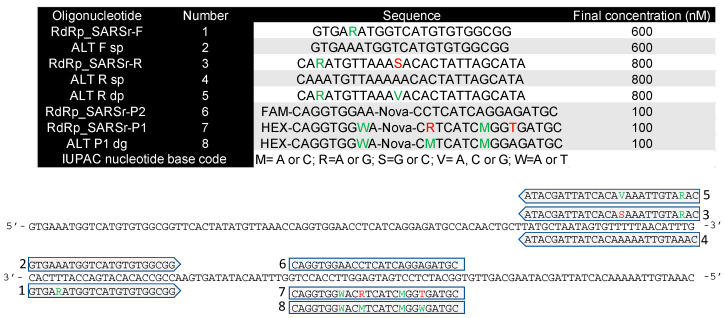
Sequence, concentration and location of oligonucleotides used in this study. Matched and mismatched nucleotides are shown in green or red, respectively. The final concentration of the reverse primer was doubled in one set of experiments.

This study used five oligonucleotide primers and the two RdRp gene probes (SARS RdRp_SARSr-P1 and SARS RdRp_SARSr-P2) from the Drosten paper, as well as a probe correcting the mistakes in the RdRp_SARSr-P1 probe. All were HPLC-purified, and their sequences and locations on the 100-bp amplicon are shown in Figure 8, with the IUPAC nucleotide codes indicating the wobble bases highlighted.

**Figure 9:**
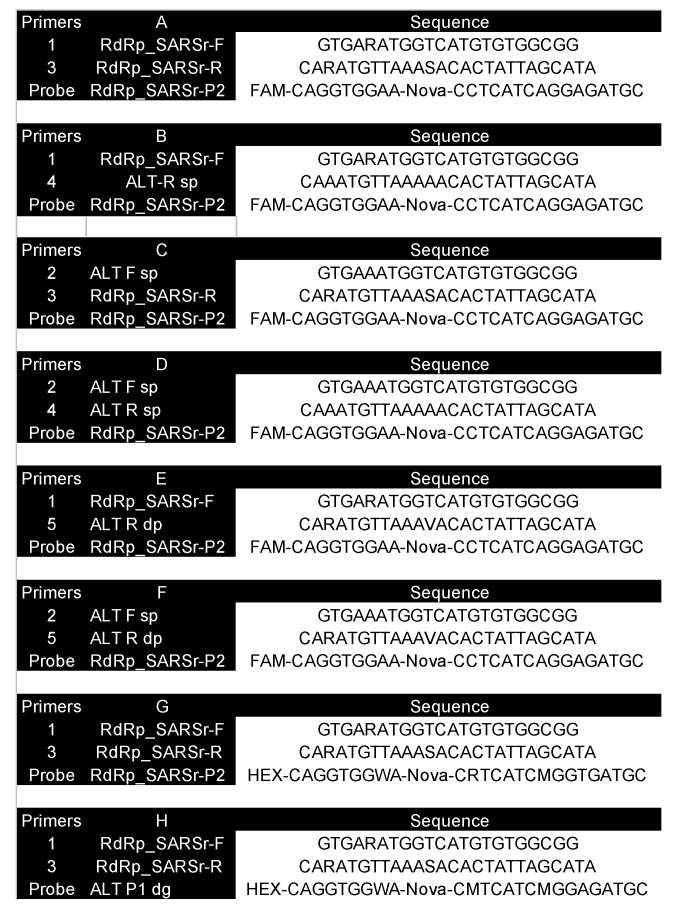
Assays A–H. (**A**) original assay; (**B**) original forward and correct specific reverse primers; (**C**) correct specific forward and original reverse primers; (**D**) correct specific primers; (**E**) original forward and correct wobble reverse primers; (**F**) correct specific forward and wobble reverse primers.

Various combinations of primers and probes were used to prepare eight assays, designated A–H (Figure 9).

### 4.2. Instruments

The two qPCR instruments used were Cole-Parmer’s PCRMax instrument (WZ-93947-00 with 48-well plates (WZ-93947-99) and Bio-Rad’s CFX Connect (1855200) with white qPCR plates (HSS9665) sealed using qPCR plate heat seals (1814030).

### 4.3. RNA

Multiple SARS-CoV-2 RNA samples were extracted from a Seracare Accuplex SARS-CoV-2 Full-Genome verification panel (505-0168) using Qiagen’s QIAamp Viral RNA mini kit (52904). The RNA quality and integrity were assessed using an Agilent 2100 Bioanalyser (G2939BA), and the samples were stored at −80 °C.

### 4.4. RT-qPCR Reactions

#### 4.4.1. RT Reactions

The SARS-CoV-2 RNA was reverse-transcribed in 20 µL using Thermo Fisher’s Superscript IV Vilo (SS4) (11756050) or Bio-Rad’s iScript (1708891), both of which use a combination of random hexamers and oligo-dT to prime cDNA synthesis. The RT conditions were 5 min at 25 °C, 5 min at 55 °C (SSIV) or 46 °C (iScript) and 5 min at 85 °C (SSIV) or 5 min at 85 °C (iScript). The cDNA samples were diluted to 50 µL with RNase-free water (Fisher Scientific 15992440).

In addition, the SARS-CoV-2 RNA was reverse-transcribed in 20 µL using PCRBio’s UltraScript (PB30.12-01) with 5 µM as the final concentration of hexamers. The RT conditions were 5 min at 25 °C, 5 min at 42 °C and 10 min at 85 °C. The cDNA sample was diluted to 50 µL with water.

#### 4.4.2. 1-Step RT-qPCR

The SARS-CoV-2 RNA was subjected to RT-qPCR amplification with the eight assays listed in Figure 9 using one or more of six commercial one-step master mixes: PCRBio 1-step Go (PB10.53-10), Takara PrimeScript (RR600A), NEB Luna (E3005S), Eurofins GSD Novaprime (PCOV6033), Quanta XLT Toughmix (95132-500) and ABI TaqPath (A15299). All reactions were carried out in 5-µL reactions using the oligonucleotide concentrations shown in Figure 8. The accuracy and reproducibility of such a small reaction volume is demonstrated in Supplementary Data File, Sheet 10. The protocol consisted of an RT (5 min at 50 °C) and an activation/denaturation step (2 min at 95 °C), followed by 40 cycles of 5 s at 95 °C and 10 s at 60 °C.

#### 4.4.3. 2-Step RT-qPCR

One-microliter aliquots of cDNA synthesised by SS4 or iScript were amplified with the six primer combinations using Meridian Bioscience SensiFast probe master mix (BIO-86050). The PCR conditions were 2 min at 95 °C, followed by 40 cycles of 5 s at 95 °C and 10 s at 60 °C. Where indicated, additional qPCR master mixes from Roche (KK4701), PrimerDesign (oasig-standard-150) and ABI (4444556) were used.

#### 4.4.4. Gradient RT-qPCR

The effect of varying the RT temperatures was tested for all six assays using the GSD Novaprime 1-step RT-qPCR master mix. A premix of RNA in 1× master mix was added to six tubes containing the various primer combinations and probes and run on a Bio-Rad CFX Connect using the following conditions: 5 min at 25 °C; 10 min at eight different temperatures (40.0 °C, 40.6 °C, 42.0 °C, 43.9 °C, 46.3 °C, 48.3 °C, 49.4 °C and 50 °C); 2 min at 95 °C and 40 cycles of 5 s at 95 °C and 10 s at 60 °C. Assays A, B and D were also analysed using the PCRBio and Quanta Toughmix 1-step reagents with the same conditions.

#### 4.4.5. Gradient qPCR

The effect of lowering the annealing temperature for the first three PCR cycles was analysed by amplifying the cDNA samples using either SYBR Green or RdRp_SARSr-P2 as reported with the appropriate SensiFast master mix: Meridian Bioscience SYBR master mix (BIO-98020) or the SensiFast probe master mix. The protocol in a Bio-Rad CFX Connect was 1-min activation at 95 °C; three cycles of 3 s at 95 °C and 10 s at 45.0 °C, 46.0 °C, 48.0 °C, 50.8 °C, 54.6 °C, 57.5 °C, 50.1 °C and 60.0 °C, followed by 36 cycles of 5 s at 95 °C and 10 s at 60 °C and a melt curve from 65 to 95 °C.

## Data Availability

All data are included in the Supplementary Data File.

## Supplementary Materials

The following are available online at https://www.mdpi.com/article/10.3390/ijms22168702/s1.

## References

[1] Bustin S.A. (2000). Absolute quantification of mRNA using real-time reverse transcription polymerase chain reaction assays. J. Mol. Endocrinol..

[2] Bustin S.A., Dorudi S. (1998). Molecular assessment of tumour stage and disease recurrence using PCR-based assays. Mol. Med. Today.

[3] Bustin S.A. (2002). Quantification of mRNA using real-time reverse transcription PCR (RT-PCR): Trends and problems. J. Mol. Endocrinol..

[4] Bustin S.A., Nolan T. (2004). Pitfalls of quantitative real-time reverse-transcription polymerase chain reaction. J. Biomol. Tech..

[5] Bustin S.A., Benes V., Nolan T., Pfaffl M.W. (2005). Quantitative real-time RT-PCR—A perspective. J. Mol. Endocrinol..

[6] Bustin S.A., Mueller R. (2005). Real-time reverse transcription PCR (qRT-PCR) and its potential use in clinical diagnosis. Clin. Sci. (Lond.).

[7] Bustin S.A. (2005). Real-time, fluorescence-based quantitative PCR: A snapshot of current procedures and preferences. Expert Rev. Mol. Diagn..

[8] Nolan T., Hands R.E., Bustin S.A. (2006). Quantification of mRNA using real-time RT-PCR. Nat. Protoc..

[9] Bustin S.A. (2008). Real-time polymerase chain reaction—Towards a more reliable, accurate and relevant assay. Eur. Pharm. Rev..

[10] Bustin S.A. (2008). Real-time quantitative PCR-opportunities and pitfalls. Eur. Pharm. Rev..

[11] Murphy J., Bustin S.A. (2009). Reliability of real-time reverse-transcription PCR in clinical diagnostics: Gold standard or substandard?. Expert Rev. Mol. Diagn..

[12] Bustin S.A., Benes V., Garson J.A., Hellemans J., Huggett J., Kubista M., Mueller R., Nolan T., Pfaffl M.W., Shipley G.L. (2009). The MIQE guidelines: Minimum information for publication of quantitative real-time PCR experiments. Clin. Chem..

[13] Bustin S.A., Beaulieu J.-F., Huggett J., Jaggi R., Kibenge F.S., Olsvik P.A., Penning L.C., Toegel S. (2010). MIQE precis: Practical implementation of minimum standard guidelines for fluorescence-based quantitative real-time PCR experiments. BMC Mol. Biol..

[14] Bustin S.A., Benes V., Garson J.A., Hellemans J., Huggett J., Kubista M., Mueller R., Nolan T., Pfaffl M.W., Shipley G.L. (2011). Primer sequence disclosure: A clarification of the MIQE guidelines. Clin. Chem..

[15] Bustin S.A., Benes V., Garson J., Hellemans J., Huggett J., Kubista M., Mueller R., Nolan T., Pfaffl M.W., Shipley G. (2013). The need for transparency and good practices in the qPCR literature. Nat. Methods.

[16] Bustin S., Nolan T. (2017). Talking the talk, but not walking the walk: RT-qPCR as a paradigm for the lack of reproducibility in molecular research. Eur. J. Clin. Investig..

[17] Sanders R., Bustin S., Huggett J., Mason D. (2018). Improving the standardization of mRNA measurement by RT-qPCR. Biomol. Detect. Quantif..

[18] Bustin S. (2017). The continuing problem of poor transparency of reporting and use of inappropriate methods for RT-qPCR. Biomol. Detect. Quantif..

[19] Naber S.P. (1994). Molecular pathology—Diagnosis of infectious disease. N. Engl. J. Med..

[20] Greub G., Sahli R., Brouillet R., Jaton K. (2016). Ten years of R&D and full automation in molecular diagnosis. Future Microbiol..

[21] Read S.J., Burnett D., Fink C.G. (2000). Molecular techniques for clinical diagnostic virology. J. Clin. Pathol..

[22] Mackay I.M., Arden K.E., Nitsche A. (2002). Real-time PCR in virology. Nucleic Acids Res..

[23] Espy M., Uhl J., Sloan L., Buckwalter S., Jones M., Vetter E., Yao J., Wengenack N., Rosenblatt J., Cockerill F. (2006). Real-time PCR in clinical microbiology: Applications for routine laboratory testing. Clin. Microbiol. Rev..

[24] Emmadi R., Boonyaratanakornkit J.B., Selvarangan R., Shyamala V., Zimmer B.L., Williams L., Bryant B., Schutzbank T., Schoonmaker M.M., Wilson J.A.A. (2011). Molecular methods and platforms for infectious diseases testing a review of FDA-approved and cleared assays. J. Mol. Diagn..

[25] Cobo F. (2012). Application of molecular diagnostic techniques for viral testing. Open Virol. J..

[26] Reta D.H., Tessema T.S., Ashenef A.S., Desta A.F., Labisso W.L., Gizaw S.T., Abay S.M., Melka D.S., Reta F.A. (2020). Molecular and Immunological Diagnostic Techniques of Medical Viruses. Int. J. Microbiol..

[27] Bustin S., Mueller R., Shipley G., Nolan T. (2021). COVID-19 and Diagnostic Testing for SARS-CoV-2 by RT-qPCR-Facts and Fallacies. Int. J. Mol. Sci..

[28] Bustin S.A., Nolan T. (2020). RT-qPCR Testing of SARS-CoV-2: A Primer. Int. J. Mol. Sci..

[29] Cuong H.Q., Hai N.D., Linh H.T., Anh N.H., Hieu N.T., Thang C.M., Thao N.T.T., Lan P.T. (2020). Comparison of Primer-Probe Sets among Different Master Mixes for Laboratory Screening of Severe Acute Respiratory Syndrome Coronavirus 2 (SARS-CoV-2). BioMed Res. Int..

[30] Bezier C., Anthoine G., Charki A. (2020). Reliability of real-time RT-PCR tests to detect SARS-Cov-2: A literature review. Int. J. Metrol. Qual. Eng..

[31] Fischer C., Mögling R., Melidou A., Kühne A., Oliveira-Filho E.F., Wolff T., Reiche J., Broberg E., Drosten C., Meijer A. (2021). Variable sensitivity in molecular detection of SARS-CoV-2 in European Expert Laboratories: External Quality Assessment, June–July 2020. J. Clin. Microbiol..

[32] Artesi M., Bontems S., Göbbels P., Franckh M., Maes P., Boreux R., Meex C., Melin P., Hayette M.-P., Bours V. (2020). A Recurrent Mutation at Position 26340 of SARS-CoV-2 Is Associated with Failure of the E Gene Quantitative Reverse Transcription-PCR Utilized in a Commercial Dual-Target Diagnostic Assay. J. Clin. Microbiol..

[33] Corman V.M., Landt O., Kaiser M., Molenkamp R., Meijer A., Chu D.K., Bleicker T., Brünink S., Schneider J., Schmidt M.L. (2020). Detection of 2019 novel coronavirus (2019-nCoV) by real-time RT-PCR. Eurosurveillance.

[34] Pillonel T., Scherz V., Jaton K., Greub G., Bertelli C. (2020). Letter to the editor: SARS-CoV-2 detection by real-time RT-PCR. Eurosurveillance.

[35] Jung Y., Park G.-S., Moon J.H., Ku K., Beak S.-H., Lee C.-S., Kim S., Park E.C., Park D., Lee J.-H. (2020). Comparative Analysis of Primer-Probe Sets for RT-qPCR of COVID-19 Causative Virus (SARS-CoV-2). ACS Infect. Dis..

[36] Nalla A.K., Casto A.M., Huang M.-L.W., Perchetti G.A., Sampoleo R., Shrestha L., Wei Y., Zhu H., Jerome K.R., Greninger A.L. (2020). Comparative Performance of SARS-CoV-2 Detection Assays Using Seven Different Primer-Probe Sets and One Assay Kit. J. Clin. Microbiol..

[37] Vogels C.B., Brito A.F., Wyllie A.L., Fauver J.R., Ott I.M., Kalinich C.C., Petrone M.E., Casanovas-Massana A., Muenker M.C., Moore A.J. (2020). Analytical sensitivity and efficiency comparisons of SARS-CoV-2 RT-qPCR primer-probe sets. Nat. Microbiol..

[38] Etievant S., Bal A., Escuret V., Brengel-Pesce K., Bouscambert M., Cheynet V., Generenaz L., Oriol G., Destras G., Billaud G. (2020). Performance Assessment of SARS-CoV-2 PCR Assays Developed by WHO Referral Laboratories. J. Clin. Med..

[39] Bustin S., Coward A., Sadler G., Teare L., Nolan T. (2020). CoV2-ID, a MIQE-compliant sub-20-min 5-plex RT-PCR assay targeting SARS-CoV-2 for the diagnosis of COVID-19. Sci. Rep..

[40] Pulsinelli G.A., Temin H.M. (1994). High rate of mismatch extension during reverse transcription in a single round of retrovirus replication. Proc. Natl. Acad. Sci. USA.

[41] Perrino F.W., Preston B.D., Sandell L.L., Loeb L.A. (1989). Extension of mismatched 3′ termini of DNA is a major determinant of the infidelity of human immunodeficiency virus type 1 reverse transcriptase. Proc. Natl. Acad. Sci. USA.

[42] Bakhanashvili M., Hizi A. (1993). The fidelity of the reverse transcriptases of human immunodeficiency viruses and murine leukemia virus, exhibited by the mispair extension frequencies, is sequence dependent and enzyme related. FEBS Lett..

[43] Yu H., Goodman M.F. (1992). Comparison of HIV-1 and avian myeloblastosis virus reverse transcriptase fidelity on RNA and DNA templates. J. Biol. Chem..

[44] Persson S., Karlsson M., Borsch-Reniers H., Ellström P., Eriksson R., Simonsson M. (2019). Missing the Match Might Not Cost You the Game: Primer-Template Mismatches Studied in Different Hepatitis A Virus Variants. Food Environ. Virol..

[45] Christopherson C., Sninsky J., Kwok S. (1997). The effects of internal primer-template mismatches on RT-PCR: HIV-1 model studies. Nucleic Acids Res..

[46] Mendelman L.V., Petruska J., Goodman M.F. (1990). Base mispair extension kinetics. Comparison of DNA polymerase alpha and reverse transcriptase. J. Biol. Chem..

[47] Stadhouders R., Pas S.D., Anber J., Voermans J., Mes T.H., Schutten M. (2010). The effect of primer-template mismatches on the detection and quantification of nucleic acids using the 5′ nuclease assay. J. Mol. Diagn..

[48] SantaLucia J.J., Hicks D. (2004). The thermodynamics of DNA structural motifs. Annu. Rev. Biophys. Biomol. Struct..

[49] Huang M.M., Arnheim N., Goodman M.F. (1992). Extension of base mispairs by Taq DNA polymerase: Implications for single nucleotide discrimination in PCR. Nucleic Acids Res..

[50] Creighton S., Huang M.M., Cai H., Arnheim N., Goodman M.F. (1992). Base mispair extension kinetics. Binding of avian myeloblastosis reverse transcriptase to matched and mismatched base pair termini. J. Biol. Chem..

[51] Mendelman L.V., Boosalis M.S., Petruska J., Goodman M.F. (1989). Nearest neighbor influences on DNA polymerase insertion fidelity. J. Biol. Chem..

[52] Newton C., Graham A., Heptinstall L., Powell S., Summers C., Kalsheker N., Smith J., Markham A. (1989). Analysis of any point mutation in DNA. The amplification refractory mutation system (ARMS). Nucleic Acids Res..

[53] Ehlen T., Dubeau L. (1989). Detection of ras point mutations by polymerase chain reaction using mutation-specific, inosine-containing oligonucleotide primers. Biochem. Biophys. Res. Commun..

[54] Wu D.Y., Ugozzoli L., Pal B.K., Wallace R.B. (1989). Allele-specific enzymatic amplification of beta-globin genomic DNA for diagnosis of sickle cell anemia. Proc. Natl. Acad. Sci. USA.

[55] Rejali N.A., Moric E., Wittwer C.T. (2018). The Effect of Single Mismatches on Primer Extension. Clin. Chem..

[56] Okayama H., Curiel D.T., Brantly M.L., Holmes M.D., Crystal R.G. (1989). Rapid, nonradioactive detection of mutations in the human genome by allele-specific amplification. J. Lab. Clin. Med..

[57] Kwok S., Kellogg D., McKinney N., Spasic D., Goda L., Levenson C., Sninsky J. (1990). Effects of primer-template mismatches on the polymerase chain reaction: Human immunodeficiency virus type 1 model studies. Nucleic Acids Res..

[58] Bru D., Martin-Laurent F., Philippot L. (2008). Quantification of the detrimental effect of a single primer-template mismatch by real-time PCR using the 16S rRNA gene as an example. Appl. Environ. Microbiol..

[59] Lefever S., Pattyn F., Hellemans J., Vandesompele J. (2013). Single-nucleotide polymorphisms and other mismatches reduce performance of quantitative PCR assays. Clin. Chem..

[60] Wu J.H., Hong P.Y., Liu W.T. (2009). Quantitative effects of position and type of single mismatch on single base primer extension. J. Microbiol. Methods.

[61] Sanchez J.A., Pierce K.E., Rice J.E., Wangh L.J. (2004). Linear-after-the-exponential (LATE)-PCR: An advanced method of asymmetric PCR and its uses in quantitative real-time analysis. Proc. Natl. Acad. Sci. USA.

[62] Pierce K.E., Sanchez J.A., Rice J.E., Wangh L.J. (2005). Linear-After-The-Exponential (LATE)-PCR: Primer design criteria for high yields of specific single-stranded DNA and improved real-time detection. Proc. Natl. Acad. Sci. USA.

[63] Jaafar R., Aherfi S., Wurtz N., Grimaldier C., Hoang V., Colson P., Raoult D., La Scola B. (2021). Correlation between 3790 qPCR positives samples and positive cell cultures including 1941 SARS-CoV-2 isolates. Clin. Infect. Dis..

[64] Rhoads D., Peaper D.R., She R.C., Nolte F.S., Wojewoda C.M., Anderson N.W., Pritt B.S. (2021). College of American Pathologists (CAP) Microbiology Committee Perspective: Caution must be used in interpreting the Cycle Threshold (Ct) value. Clin. Infect. Dis..

[65] Bullard J., Dust K., Funk D., Strong J.E., Alexander D., Garnett L., Boodman C., Bello A., Hedley A., Schiffman Z. (2020). Predicting infectious severe acute respiratory syndrome coronavirus 2 from diagnostic samples. Clin. Infect. Dis..

[66] Arons M.M., Hatfield K.M., Reddy S.C., Kimball A., James A., Jacobs J.R., Taylor J., Spicer K., Bardossy A.C., Oakley L.P. (2020). Presymptomatic SARS-CoV-2 Infections and Transmission in a Skilled Nursing Facility. N. Engl. J. Med..

[67] La Scola B., Le Bideau M., Andreani J., Grimaldier C., Colson P., Gautret P., Raoult D. (2020). Viral RNA load as determined by cell culture as a management tool for discharge of SARS-CoV-2 patients from infectious disease wards. Eur. J. Clin. Microbiol. Infect. Dis..

[68] Dahdouh E., Lázaro-Perona F., Romero-Gomez M.P., Mingorance J., Garcia-Rodriguez J. (2021). Ct values from SARS-CoV-2 diagnostic PCR assays should not be used as direct estimates of viral load. J. Infect..

[69] Tom M.R., Mina M.J. (2020). To interpret the SARS-CoV-2 test, consider the cycle threshold value. Clin. Infect. Dis..

[70] Shah S., Singhal T., Davar N., Thakkar P. (2021). No correlation between Ct values and severity of disease or mortality in patients with COVID 19 disease. Indian J. Med. Microbiol..

[71] Trunfio M., Venuti F., Alladio F., Longo B.M., Burdino E., Cerutti F., Ghisetti V., Bertucci R., Picco C., Bonora S. (2021). Diagnostic SARS-CoV-2 Cycle Threshold Value Predicts Disease Severity, Survival, and Six-Month Sequelae in COVID-19 Symptomatic Patients. Viruses.

[72] Basso D., Aita A., Navaglia F., Franchin E., Fioretto P., Moz S., Bozzato D., Zambon C.-F., Martin B., Dal Prà C. (2020). SARS-CoV-2 RNA identification in nasopharyngeal swabs: Issues in pre-analytics. Clin. Chem. Lab. Med..

[73] Binnicker M.J. (2020). Challenges and Controversies to Testing for COVID-19. J. Clin. Microbiol..

[74] Carroll A., McNamara E. (2021). Comparison and correlation of commercial SARS-CoV-2 real-time-PCR assays, Ireland, June 2020. Eurosurveillance.

[75] Aquino-Jarquin G. (2021). The Raw Cycle Threshold Values from Reverse-transcription Polymerase Chain Reaction Detection Are Not Viral Load Quantitation Units. Clin. Infect. Dis..

[76] Romero-Gómez M.P., Gómez-Sebastian S., Cendejas-Bueno E., Montero-Vega M.D., Mingorance J., García-Rodríguez J., Group S.-C.-W. (2021). Ct value is not enough to discriminate patients harbouring infective virus. J. Infect..

