# Supplementary material for: RT-qPCR Diagnostics: The “Drosten” SARS-CoV-2 Assay Paradigm
Source: Int J Mol Sci. 2021 Aug 13;22(16):8702. doi: 10.3390/ijms22168702 (PMC8395416; doi:10.3390/ijms22168702)
Supplement: Supplementary file 1 [file ijms-22-08702-s001.zip › ijms-1327107.figure S1.pdf]

A

	TaqPath	GSD	Quanta	PrimeScript	Luna	Biorad	Sigma	PCRBio	Sansure
Cq1	23.53	23.24	24.29	24.41	24.90	25.49	26.38	24.39	25.32
Cq2	23.00	23.25	24.00	23.97	24.97	25.35	26.36	24.07	25.19
Cq3	22.81	23.17	24.01	24.36	24.59	24.84	26.21	24.19	25.19
Cq4	23.88	23.87	25.19	24.68	24.80	24.85	26.63	24.05	25.05
Cq5	23.35	23.53	24.70	25.91	25.49	25.65	27.04	24.69	25.24
Average	**23.31**	**23.41**	**24.44**	**24.67**	**24.95**	**25.24**	**26.53**	**24.28**	**25.20**
Min	22.81	23.17	24.00	23.97	24.59	24.84	26.21	24.05	25.05
Max	23.88	23.87	25.19	25.91	25.49	25.65	27.04	24.69	25.32
ΔCq	1.07	0.70	1.19	1.94	0.91	0.80	0.84	0.64	0.27
Fold-difference	2.10	1.62	2.29	3.85	1.87	1.75	1.79	1.55	1.21

	Overall
Average	24.67
Min	22.81
Max	27.04
ΔCq	4.23
Fold-difference	18.77

B

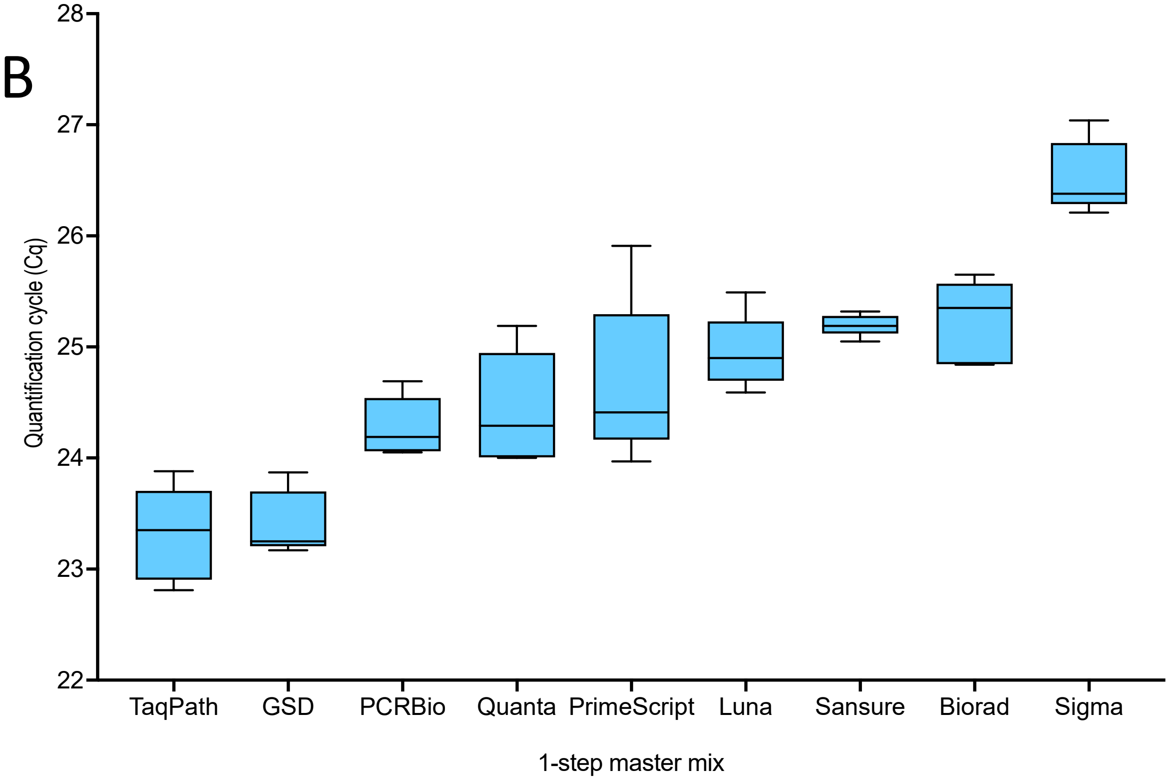

Figure S1. Performance of a commercial SARS-CoV-2 primer and probe set with nine different 1-step master mixes. (A) Quantification cycles (Cqs). (B) Plot of the Cqs ordered by the average Cq. The minimum Cq recorded is 22.81, the maximum one 27.04. This ΔCq of 4.23 corresponds to an apparent 19-fold difference for the same amount of input RNA.
